# The construction of a heterostructured RGO/g-C_3_N_4_/LaCO_3_OH composite with enhanced visible light photocatalytic activity for MO degradation†

**DOI:** 10.1039/d3ra02415f

**Published:** 2023-05-19

**Authors:** Deng Gu, Yuanjin Wang, Zhiman Liang, Yanting Dou, Zhenhe Xu, Jiqi Zheng, Yaguang Sun, Fu Ding, Yu Gao

**Affiliations:** a College of Environmental and Chemical Engineering, Dalian University Dalian 116622 China xuzh@syuct.edu.cn jiqizheng@yeah.net gaoy777@126.com; b Key Laboratory of Inorganic Molecule-Based Chemistry of Liaoning Province, Shenyang University of Chemical Technology Shenyang 110142 China dingfu@syuct.edu.cn

## Abstract

The construction of a heterojunction and the introduction of a cocatalyst can both promote the transfer of photogenerated electrons, which are effective strategies to enhance photocatalytic efficiency. In this paper, a ternary RGO/g-C_3_N_4_/LaCO_3_OH composite was synthesized by constructing a g-C_3_N_4_/LaCO_3_OH heterojunction and introducing a non-noble metal cocatalyst RGO through hydrothermal reactions. TEM, XRD, XPS, UV-vis diffuse reflectance spectroscopy, photo-electrochemistry and PL tests were carried out to characterize the structures, morphologies and carrier separation efficiencies of products. Benefiting from the boosted visible light absorption capability, reduced charge transfer resistance and facilitated photogenerated carrier separation, the visible light photocatalytic activity of the ternary RGO/g-C_3_N_4_/LaCO_3_OH composite was effectively improved, resulting in a much increased MO (methyl orange) degradation rate of 0.0326 min^−1^ compared with LaCO_3_OH (0.0003 min^−1^) and g-C_3_N_4_ (0.0083 min^−1^). Moreover, by combining the results of the active species trapping experiment with the bandgap structure of each component, the mechanism of the MO photodegradation process was proposed.

## Abstract 2

The photodegradation performance of ternary RGO/g-C_3_N_4_/LaCO_3_OH composite under visible light irradiation is enhanced by the construction of g-C_3_N_4_/LaCO_3_OH heterojunction and the introduction of RGO as a cocatalyst.

## Graphical abstract

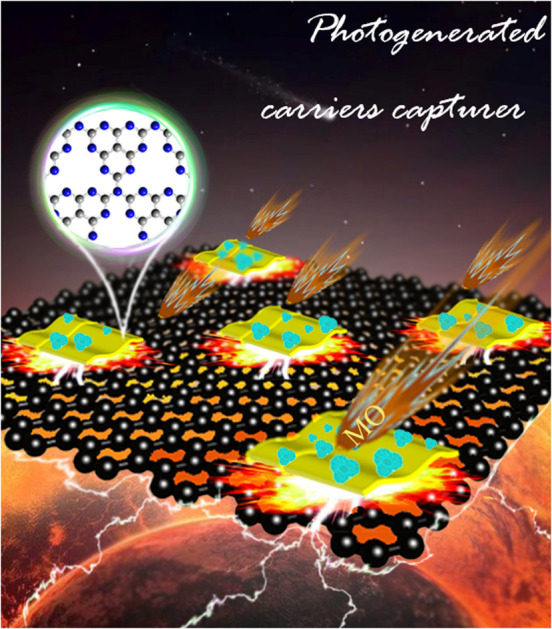

## 1. Introduction

Environmental pollution is one of the main challenges for the rapid development of a green economy.^1^ Photocatalysis based on semiconductors is considered as a promising technology with wide application prospects to address environmental problems.^2^ However the development of photocatalysts with low cost, high stability and efficiency remains a challenge.^3^ Though rare earth-based materials including YVO_4_,^4^ CeO_2_ (ref. 5) and LaCO_3_OH^6^ have attracted wide attention in photocatalysis, their application is still limited because of their large bandgaps, leading to a poor ability in visible light absorption.^7^ The construction of heterojunctions,^8,9^ the introduction of cocatalysts^10^ and elemental doping^11^ are effective strategies to decrease the bandgaps of rare earth photocatalysts and hence improve the utilization efficiency of visible light and accelerate the kinetics of photocatalytic process.

LaCO_3_OH is a rare earth hydroxyl carbonate with an excellent luminescence property, showing a great potential in photocatalytic pollutants degradation and H_2_ evolution.^12,13^ LaCO_3_OH has a relatively broad bandgap of 4.0 eV, which could inhibit the recombination of photogenerated carriers.^14^ On the other side, as a result of the broad bandgap, it can only absorb ultraviolet light in photocatalytic reactions. Based on recent research, the visible light absorption capability of LaCO_3_OH can be boosted by constructing heterojunction.^15^ Under visible light irradiation, pure LaCO_3_OH showed a negligible photocatalytic activity, while heterostructured g-C_3_N_4_/LaCO_3_OH synthesized through a one-step hydrothermal reaction exhibited a substantially enhanced NO removal ratio of 30.3% after 30 min.^16^ g-C_3_N_4_, a kind of semiconductor photocatalyst, has drawn wide attention in vast fields because of its visible light response and layered structure, which can provide rich surface chemistry and shorten the electron transfer pathways.^17^ While there are only few reports about g-C_3_N_4_/LaCO_3_OH heterojunction as the catalyst for the photodegradation of organic dyes at present. Wang *et al.*^13^ synthesized a g-C_3_N_4_/LaCO_3_OH heterostructure for photocatalytic NO removal, which showed an improved NO removal ratio of 30.3% compared with pristine g-C_3_N_4_ (19.3%) after 30 min visible light irradiation. Similar composite also shown considerable improvement in N_2_ photofixation (8.2 mM g^−1^ h^−1^) compared to g-C_3_N_4_ (2.1 mM g^−1^ h^−1^).^18^ While there is still no report about the application of g-C_3_N_4_/LaCO_3_OH heterojunction in the photodegradation of dyes. The introduction of cocatalyst such as noble metal (Pt^19^ and Ag^20^), non-noble metal (Ni^21^) and non-metal material such as graphene oxide (GO) can restrain the recombination of photogenerated carriers and enhance visible light absorption capacity.^22^ Among all these cocatalysts, GO has fascinating properties including good conductivity, high carrier mobility and large exposed specific surface, which can provide abundant active sites for the photocatalytic process.^23^ Therefore, for the first time, we proposed to introduce reduced graphene oxide (RGO) into g-C_3_N_4_/LaCO_3_OH heterojunction and result in a heterostructured ternary composite for photocatalytic degradation of the organic dye. Benefitting from the extended light absorption in visible light region along with accelerated separation kinetics of charge carriers, the constructed RGO/g-C_3_N_4_/LaCO_3_OH composite can serve as the catalyst for the photodegradation of methyl orange (MO) under visible light irradiation with a superior performance than each component.

Herein, ternary composite RGO/g-C_3_N_4_/LaCO_3_OH heterojunction was synthesized through hydrothermal reactions and its photocatalytic activity was evaluated by using it as the catalyst in the degradation of MO under visible light irradiation. The photocatalysis mechanism was investigated and the migration pathway of photogenerated carriers was proposed. This work is excepted to provide a novel strategy to enhance the photocatalytic activity of rare-earth based semiconductors with broad bandgaps for the photodegradation of organic dyes.

## 2. Experimental section

### 2.1 Materials

Analytical grade chemicals were used directly in the experiments. Graphite powder, K_2_S_2_O_8_, H_2_SO_4_, HCl, KMnO_4_ and BaCl_2_ were purchased from Sinopharm, China. Urea, La(NO_3_)_3_·6H_2_O and MO were provided by Aladdin, China.

g-C_3_N_4_ was synthesized through the method in the literature.^24^ 10.0 g urea was calcined at 250 °C for 1 h, then 350 °C for 2 h and 550 °C for another 2 h in a tube furnace. The obtained pale yellow powder was washed with distilled water then dried at 60 °C for 12 h.

*X* wt% g-C_3_N_4_/LaCO_3_OH (*X* represents the mass ratio of g-C_3_N_4_, *X* = 15.0, 25.0, 35.0) was synthesized through a hydrothermal reaction. For 15.0 wt% g-C_3_N_4_/LaCO_3_OH, 50.0 mg g-C_3_N_4_ and 671.6 mg La(NO_3_)_3_·6H_2_O were dispersed in 35 mL distilled water and stirred for 3 h. After adjusting pH to 8.0–8.5 using 0.1 M NaOH aqueous solution, the obtained suspension was transferred to 100 mL autoclave and heated at 160 °C for 6 h. After washed with distilled water for 3 times, the product was dried at 60 °C for 12 h. Pure LaCO_3_OH was also produced *via* a reported method for comparison.^14^

**Scheme 1:**
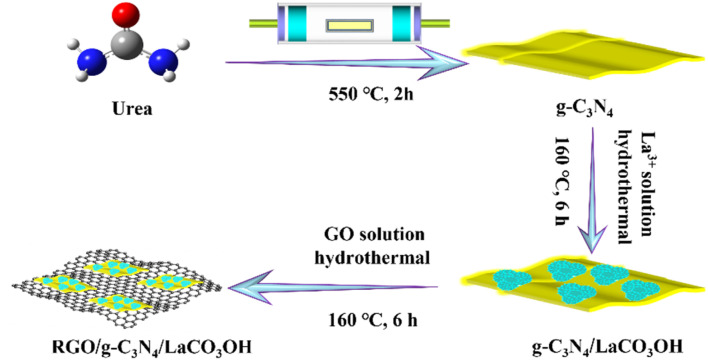
The schematic fabrication process of RGO/g-C_3_N_4_/LaCO_3_OH composite.

RGO/g-C_3_N_4_/LaCO_3_OH was obtained through a hydrothermal reaction using g-C_3_N_4_/LaCO_3_OH and GO as raw materials, as shown in Scheme 1, and the latter was produced by the Hummers' method.^10^ For *Y* wt% RGO/g-C_3_N_4_/LaCO_3_OH (*Y* represents the mass ratio of RGO, *X* = 0.6, 0.8, 1.0), 50.0 mg 25.0 wt% g-C_3_N_4_/LaCO_3_OH was dispersed in 35.0 mL distilled water, in which 400.0 μL GO suspension (1.0 mg mL^−1^) was added. The obtained mixture was heated at 160 °C for 6 h in an autoclave, and the product was washed and dried in the same way as the synthesis of g-C_3_N_4_/LaCO_3_OH sample.

### 2.2 Characterization

An X-ray diffractometer (XRD, Bruker D8 Advance) with Cu Kα radiation was used to characterize the products' structures. X-ray photoelectron spectroscopy (XPS) was performed on Thermal ESCALAB 250Xi. The morphologies, microstructures, elemental mappings and the corresponding energy-dispersive X-ray spectroscopy (EDS) of the products were recorded by transmission electron microscopy (TEM, JEOL JEM 2010 EX). UV-vis diffuse reflectance spectra were tested on a Shimadzu UV-2500 spectrophotometer using BaSO_4_ as the reference. Photoluminescence spectra (PL) were characterized by a F4500 Hitachi spectrophotometer with an excitation wavelength of 330 nm. The photoelectrochemical performance and electrochemical impedance spectroscopy (EIS) of the products were performed on a CHI 660E workstation using a three electrode system in 0.2 M Na_2_SO_4_ aqueous solution. The counter electrode was Pt wire and the reference electrode was saturated calomel electrode. For the working electrode, 10 mg product was dispersed in 1.0 mL ethanol and the suspension was dropped on fluorine-doped tin oxide (FTO) glass.

### 2.3 Photocatalytic tests

Photocatalytic performances of the obtained products were evaluated through the degradation of MO under visible light irradiation (*λ* > 420 nm), which was provided by a Xe lamp with a filter. The photograph of the lamp and the transmission spectrum of the filtered light were shown in Fig. S1.† 10 mg sample and 20 mL MO solution (10 mg L^−1^) was added in to a quartz reactor, whose temperature was maintained at 30 °C. After stirring in darkness for 1 h to establish the adsorption–desorption equilibrium, the suspension was exposed in visible light. The concentrations of MO under different irritation times were then calculated based on the intensities of the peak at 465 nm in UV-vis spectra.

## 3. Results and discussion

**Fig. 1:**
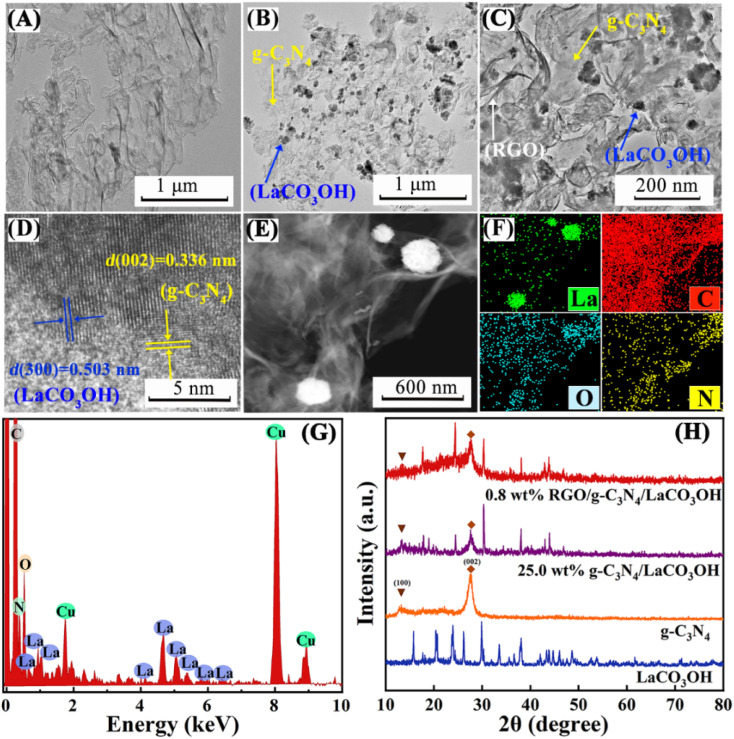
TEM images of (A) g-C_3_N_4_, (B) g-C_3_N_4_/LaCO_3_OH and (C) RGO/g-C_3_N_4_/LaCO_3_OH; a high-resolution TEM image (D), a scanning TEM image (E), the corresponding elemental mappings (F) and the EDS spectrum (G) of RGO/g-C_3_N_4_/LaCO_3_OH; (H) XRD patterns of LaCO_3_OH, g-C_3_N_4_, g-C_3_N_4_/LaCO_3_OH and RGO/g-C_3_N_4_/LaCO_3_OH.

As shown in Scheme 1, ternary RGO/g-C_3_N_4_/LaCO_3_OH heterojunction was synthesized through a two-step hydrothermal process. It can be observed in Fig. 1A that the synthesized g-C_3_N_4_ composes of 2D nanosheets with smooth and uniform surface. g-C_3_N_4_/LaCO_3_OH heterojunction was fabricated by the *in situ* growth of LaCO_3_OH on the surface of g-C_3_N_4_*via* the hydrothermal reaction. In this process, g-C_3_N_4_ served as a self-sacrificial agent to produce CO_3_^2−^ and OH^−^ due to the weaker binding energy of C chain. Then La^3+^ ions adsorbed on g-C_3_N_4_ reacted with the generated CO_3_^2−^ and OH^−^ to produced LaCO_3_OH, resulting in the *in situ* construction of g-C_3_N_4_/LaCO_3_OH heterojunction.^13,18^ The TEM image (Fig. 1B) shows that LaCO_3_OH nanoparticles with diameters of 20–50 nm are evenly dispersed on g-C_3_N_4_. Then RGO was further introduced as the cocatalyst to obtain ternary RGO/g-C_3_N_4_/LaCO_3_OH (Fig. 1C). In the high-resolution TEM image, the heterostructure interface between the (3 0 0) plane of LaCO_3_OH and (0 0 2) plane of g-C_3_N_4_ can be clearly observed (Fig. 1D), suggesting the construction of g-C_3_N_4_/LaCO_3_OH heterojunction. The scanning TEM image, corresponding elemental mappings and EDS spectrum (Fig. 1E–G) verify that RGO/g-C_3_N_4_/LaCO_3_OH are composed of La, C, O and N element, and La mainly distributed in the nanoparticles, which is consistent with the TEM results. The XRD patterns of LaCO_3_OH, g-C_3_N_4_, g-C_3_N_4_/LaCO_3_OH and RGO/g-C_3_N_4_/LaCO_3_OH are shown in Fig. 1H. For the synthesized LaCO_3_OH, its XRD pattern agrees well with hexagonal LaCO_3_OH (JCPDS No. 49-0981).^14^ In the XRD pattern of g-C_3_N_4_, two typical peaks at 13.20° and 27.54° are corresponding to the (1 0 0) and (0 0 2) plane, respectively.^16^ The typical peaks of both LaCO_3_OH and g-C_3_N_4_ can be observed in the g-C_3_N_4_/LaCO_3_OH and RGO/g-C_3_N_4_/LaCO_3_OH composite with good crystallinities, indicating that the crystal structures of these two components were maintained during the hydrothermal processes. With the introduction of RGO, the (0 0 2) peak of g-C_3_N_4_ broaden because of the interlayer stacking between 2D RGO and g-C_3_N_4_ sheets.^25^ All the above results clarified that ternary RGO/g-C_3_N_4_/LaCO_3_OH heterojunction was successfully constructed.

**Fig. 2:**
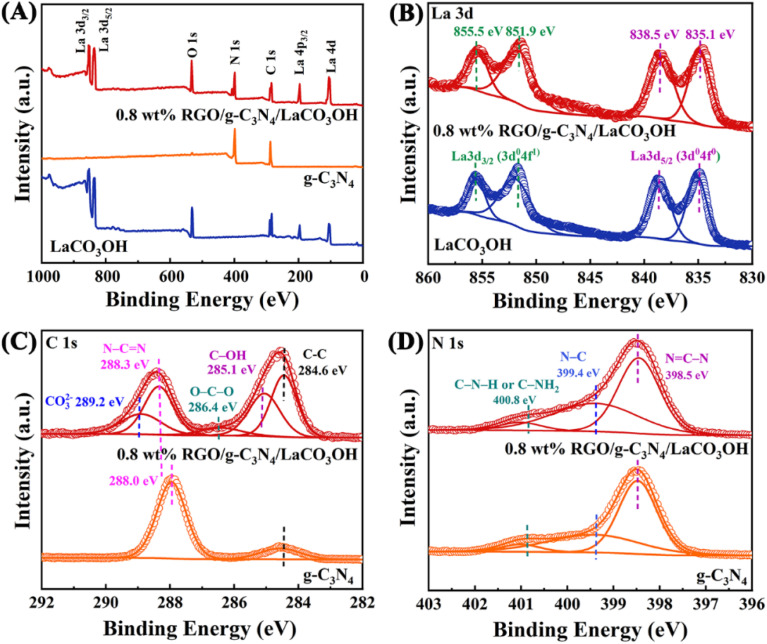
XPS spectra of RGO/g-C_3_N_4_/LaCO_3_OH: (A) the survey spectrum compared with g-C_3_N_4_ and LaCO_3_OH; (B) La 3d spectrum compared with LaCO_3_OH; (C) C 1s spectrum and (D) N 1s spectrum compared with g-C_3_N_4_.

XPS spectra are used to further study the constitution and surface chemical states of the products. In the survey spectrum of RGO/g-C_3_N_4_/LaCO_3_OH (Fig. 2A), La, C, O and N elements can be identified, which is in accordance with the results of EDS. As shown in Fig. 2B, the spectrum of La 3d can be divided into two pairs of peaks. Peaks at 851.9 and 835.1 eV can be assigned to La 3d_3/2_ and 3d_5/2_ of La^3+^, respectively, and peaks at 855.5 and 838.5 eV are two corresponding shakeup satellite peaks.^14^ C 1s spectrum of RGO/g-C_3_N_4_/LaCO_3_OH can be fitted into five peaks considering three different sources (Fig. 2C). Peaks at 286.4, 285.1 and 286.4 eV can be indexed to O–C–O, C–OH and C–C in RGO, respectively. Peaks at 289.2 and 288.3 eV stem from CO_3_^2−^ in LaCO_3_OH and N–C=N in g-C_3_N_4_, respectively. Compared with g-C_3_N_4_, the binding energy of N–C=N in RGO/g-C_3_N_4_/LaCO_3_OH increased by 0.3 eV because of the electron transfer at the interfaces of the composite.^6^ For N 1s spectrum, peaks at 400.8, 399.4 and 398.5 eV are attributed to C–N–H (or C–NH_2_), N–C and N=C–N in g-C_3_N_4_, respectively (Fig. 2D).^17^ The groups such as –OH and –NH_2_ can provide abundant active sites for the photodegradation of MO.

**Fig. 3:**
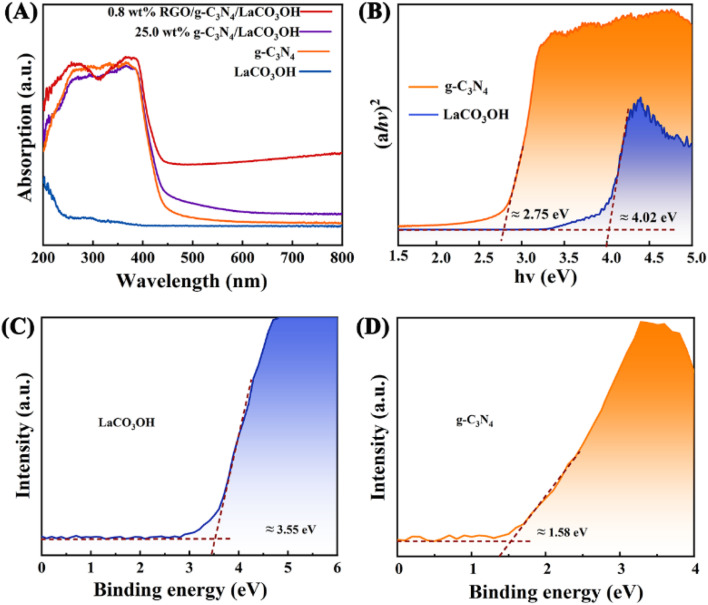
(A) The UV-vis spectra of LaCO_3_OH, g-C_3_N_4_, g-C_3_N_4_/LaCO_3_OH and RGO/g-C_3_N_4_/LaCO_3_OH; (B) the bandgap energy diagram and the valence band (XPS) spectra of the (C) LaCO_3_OH and (D) g-C_3_N_4_.

UV-vis spectra of LaCO_3_OH and g-C_3_N_4_ were measured to evaluate their band gap structures (Fig. 3A). The light absorption edge of g-C_3_N_4_ is 460 nm, indicating its small bandgap and good response to the visible light. While LaCO_3_OH exhibits a light absorption edge of 300 nm, demonstrating its large bandgap along with poor visible light absorption capability. For g-C_3_N_4_/LaCO_3_OH and RGO/g-C_3_N_4_/LaCO_3_OH, light absorption edges are located at 475 and 500 nm respectively, indicating the improved visible light absorption capability benefiting from the construction of heterojunction and the introduction of cocatalyst RGO. The band gaps *E*_g_ (eV) of LaCO_3_OH and g-C_3_N_4_ can be calculated based on the Kubelka–Munk function:

1 *αħv* = *A*(*ħv* − *E*_g_)^*n*/2^

where *A*, *v*, *ħ* and *α* are constant, light frequency, Plank's constant and the absorption coefficient, respectively. For semiconductors such as g-C_3_N_4_ and LaCO_3_OH, *n* = 4.^24^ The calculated *E*_g_ for LaCO_3_OH and g-C_3_N_4_ are 2.75 and 4.02 eV, respectively (Fig. 3B). According to the results of XPS spectra, the valence band (VB) potentials (*E*_VB_) of LaCO_3_OH and g-C_3_N_4_ can also be calculated. As shown in Fig. 3C and D, the results are 3.55 and 1.58 eV, respectively. The conduction band (CB) potentials (*E*_CB_) can be calculated by the equation:

2 *E*_CB_ = *E*_CB_ − *E*_g_

The calculated *E*_CB_ of LaCO_3_OH and g-C_3_N_4_ are 0.47 and −1.17 eV, respectively.

**Fig. 4:**
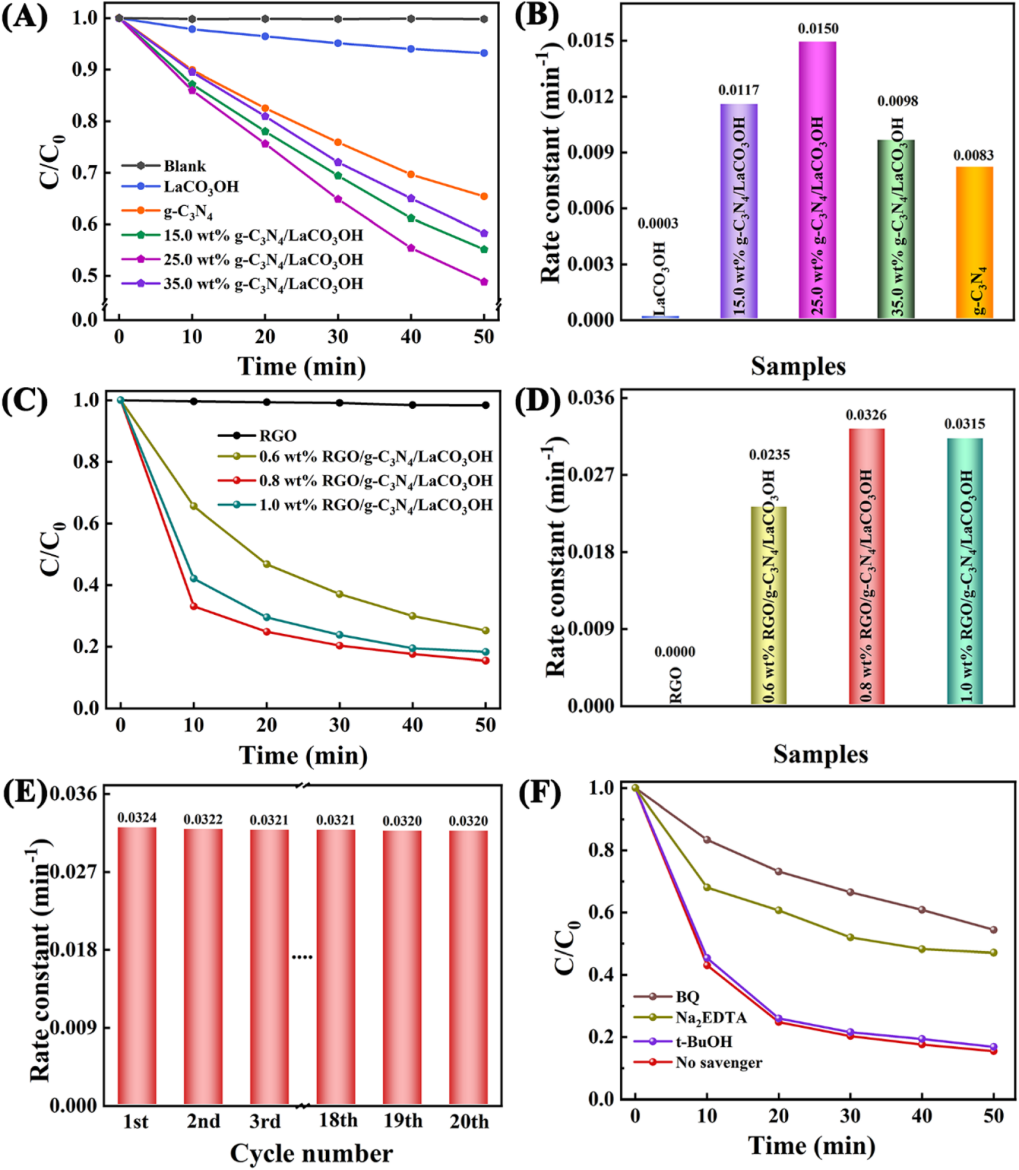
(A) The activities of the MO photodegradation using RGO, g-C_3_N_4_, LaCO_3_OH and g-C_3_N_4_/LaCO_3_OH with different ratios of g-C_3_N_4_ as catalysts during 50 min and (B) the corresponding photodegradation rate constants; (C) the activities of the MO photodegradation using RGO and RGO/g-C_3_N_4_/LaCO_3_OH with different ratios of RGO as catalysts during 50 min and (D) the corresponding photodegradation rate constants; (E) the cyclic tests and (F) activities of the MO photodegradation in the presence of three scavengers of 0.8% RGO/g-C_3_N_4_/LaCO_3_OH.

The photocatalytic performance of the obtained heterostructured products were assessed by the photodegradation of MO under the visible light irradiation. After 50 min, the degradation of MO without catalysts is negligible, as shown in Fig. 4A. For pure LaCO_3_OH, the photodegradation activity is low with a MO removal ratio of only 6.8% after 50 min because of its poor visible light absorption capability, which is proved by its UV-vis spectrum. Constructing g-C_3_N_4_/LaCO_3_OH heterojunction can significantly improve the MO removal ratio. Among the composites with different mass ratios of g-C_3_N_4_, 25% g-C_3_N_4_/LaCO_3_OH exhibited the highest activity with a calculated photodegradation rate constant of 0.0150 min^−1^ (Fig. 4B), which is 50.0 and 1.8 times higher than LaCO_3_OH and g-C_3_N_4_ respectively. Such results confirmed that the construction of heterojunction could be conducive to the improvement of photocatalytic activity compared with each component.

Whereas, though the MO removal ratio has been effectively improved, the photodegradation of MO only 51.2%. To further improve the activity of the photocatalyst and shorten the reaction time, RGO was introduced into the composite as the cocatalyst. Photocatalytic performances of ternary RGO/g-C_3_N_4_/LaCO_3_OH composite with different mass ratios of RGO is shown in Fig. 4C. Though RGO itself shows no activity in the photodegradation of MO as expected, the introduction of RGO into 25% g-C_3_N_4_/LaCO_3_OH can still further enhance its photocatalytic performance. When the reaction time decreased to 50 min, the MO removal ratio of 0.8% RGO/g-C_3_N_4_/LaCO_3_OH can be remarkably improved to 84.5%, which is the highest among ternary composites with different mass ratios, and is much improved compared with 25% g-C_3_N_4_/LaCO_3_OH (51.2%). The corresponding photodegradation rate constant of 0.8% RGO/g-C_3_N_4_/LaCO_3_OH is 0.0326 min^−1^, which is 2.17 times higher than that of 25% g-C_3_N_4_/LaCO_3_OH (Fig. 4D). Such improvement arises from the introduction of cocatalyst RGO, which can serve as the transfer medium for the photogenerated carriers, thus can accelerate their transfer kinetics and restrain their recombination, leading to an increased rate of photocatalytic reaction.

The high stability of ternary RGO/g-C_3_N_4_/LaCO_3_OH composite was clarified by the cycling test carried out using 0.8% RGO/g-C_3_N_4_/LaCO_3_OH as the catalyst. As shown in Fig. 4E, the rate constant remains basically stable during 20 cycles. The slight decrease was generated by the sample loss during the washing and drying process after each cycle. TEM images shows that after the long-time cycling, there is no obvious change in the microstructure of the RGO/g-C_3_N_4_/LaCO_3_OH composite, confirming its high structural stability (Fig. S2†). Moreover, the active species trapping experiment was taken to analyze the active species during the photocatalytic degradation process (Fig. 4F). There is no significant change on MO removal ratio with the existence of *t*-BuOH, demonstrating that ˙OH radical does not involved in the photodegradation reaction. While with the addition of 1,4-benzoquinone (BQ) and Na_2_EDTA, the MO removal ratio declined from 84.5% to 45.6% and 52.9% respectively, indicating that both active species ˙O_2_^−^ and h^+^ play a critical role in the photodegradation process. The above characterizations show that compared with single components, RGO/g-C_3_N_4_/LaCO_3_OH exhibits an enhanced photocatalytic activity for MO degradation, which is owing to the construction of heterojunction and the introduction of cocatalyst RGO.

**Fig. 5:**
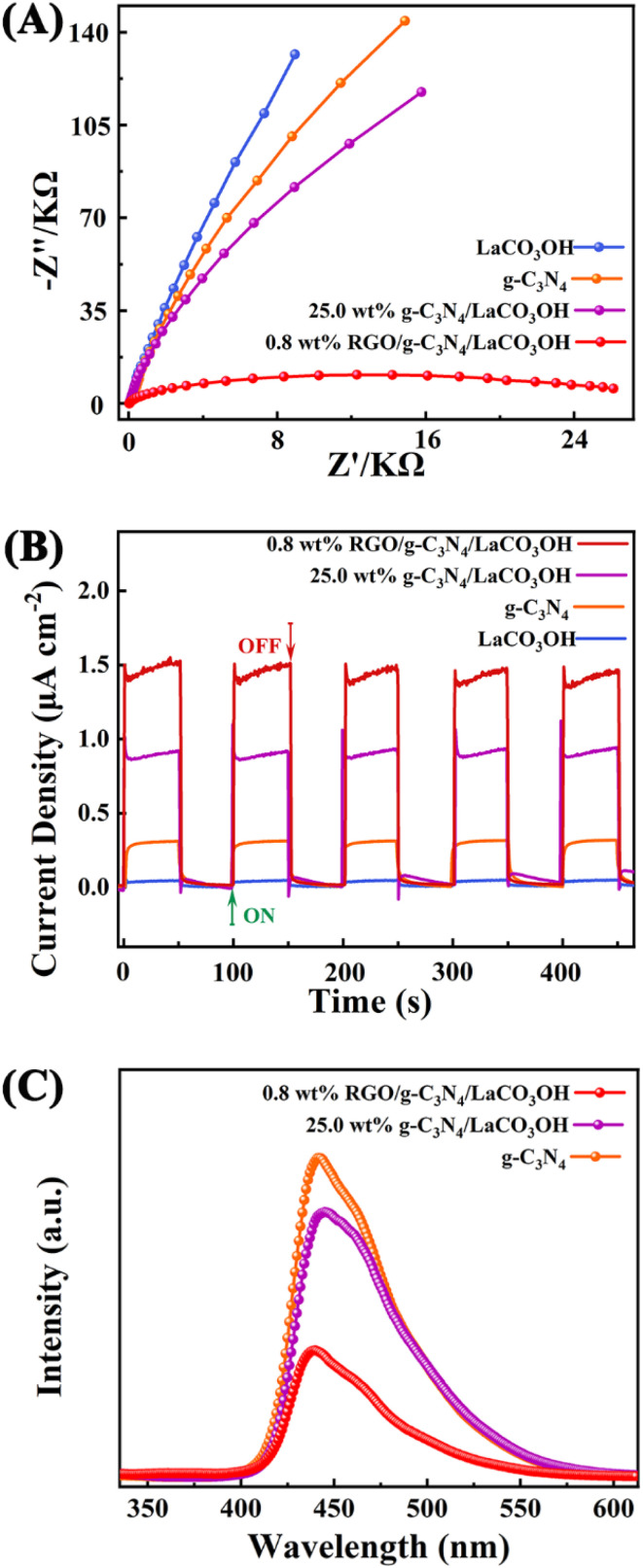
(A) The Nyquist plots of RGO, g-C_3_N_4_, LaCO_3_OH and RGO/g-C_3_N_4_/LaCO_3_OH in the frequency range from 10^6^ Hz to 0.1 Hz; (B) transient photocurrent responses and (C) the PL spectra.

More photoelectrochemical characterizations including EIS tests, transient photocurrent responses and PL spectroscopy were performed to study the separation efficiency of photogenerated charge carriers. The Nyquist plots (Fig. 5A) demonstrates that 0.8% RGO/g-C_3_N_4_/LaCO_3_OH has the smallest semicircle radius in the high-frequency region compared with that of g-C_3_N_4_/LaCO_3_OH, g-C_3_N_4_ and LaCO_3_OH, indicating its lowest charge transfer resistance benefited from the construction of the heterojunction and the introduction of RGO, leading to the highest separation efficiency of photogenerated electron–hole pairs.^26^ Transient photocurrent curves during five on/off cycles are shown in Fig. 5B. 0.8% RGO/g-C_3_N_4_/LaCO_3_OH generates a stable and highest photocurrent of 1.52 μA cm^−2^, which is 1.78 and 4 times higher compared with 25% g-C_3_N_4_/LaCO_3_OH (0.85 μA cm^−2^) and g-C_3_N_4_ (0.38 μA cm^−2^), indicating the heterostructured ternary composite can effectively accelerate the charge transfer process. In PL spectra, the lower PL intensity means the lower recombination rate and longer lifetime of the photogenerated carriers.^27^ As shown in Fig. 5C, compared with g-C_3_N_4_ and 25% g-C_3_N_4_/LaCO_3_OH, the PL intensity of 0.8% RGO/g-C_3_N_4_/LaCO_3_OH is much lower, confirming that cocatalyst RGO plays an important role in the restriction of photogenerated carriers' recombination. Overall, the synergistic effect of heterostructure and cocatalyst RGO endows RGO/g-C_3_N_4_/LaCO_3_OH composite an improved photocatalytic activity by reducing the charge transfer resistance, facilitating the separation of charge carries and lowering their recombination rate.

**Fig. 6:**
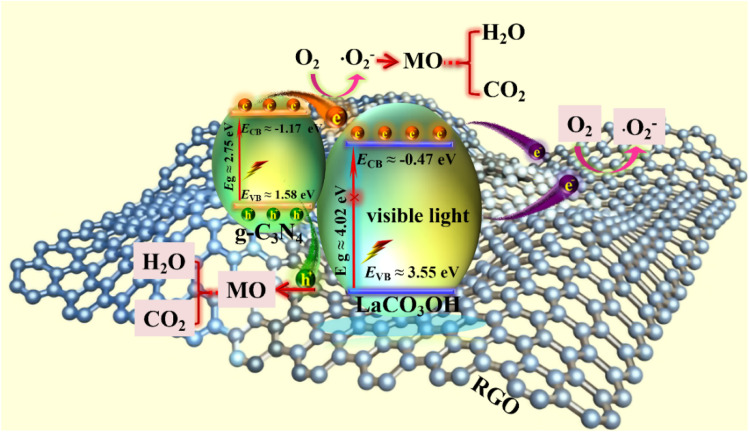
The schematic of the proposed mechanism of RGO/g-C_3_N_4_/LaCO_3_OH composites for MO photodegradation under visible light irradiation.

According to above characterizations, the mechanism of the MO photodegradation process using RGO/g-C_3_N_4_/LaCO_3_OH composite as the catalyst was proposed, as shown in Fig. 6. With the irradiation of visible light, g-C_3_N_4_ with a smaller bandgap of 2.75 eV can be excited and produce photogenerated electrons and holes, while LaCO_3_OH does not response to visible light due to its large bandgap (4.02 eV). The electrons generated on the VB of g-C_3_N_4_ (1.58 eV) can be excited and separate with holes, then transfer to the CB of g-C_3_N_4_ (−1.17 eV). As g-C_3_N_4_ has a more negative CB compared with LaCO_3_OH (−0.47 eV), the photogenerated electrons will transfer to the CB of LaCO_3_OH driven by the interfacial electric field force, thus the recombination of photogenerated electrons and holes can be retarded. The introduction of RGO as cocatalyst can not only enhance the light absorption efficiency but also provide abundant active sites for the photocatalytic reaction because of the enlarged surface area and facilitated charge transfer kinetics of RGO. The main reactions in the photodegradation process of MO are as follows:g-C_3_N_4_ + *ħv* → g-C_3_N_4_ (h^+^) + LaCO_3_OH/g-C_3_N_4_ (e^−^)LaCO_3_OH/g-C_3_N_4_ (e^−^) + *ħv* → 2D-RGO (e^−^)LaCO_3_OH/g-C_3_N_4_ (e^−^) + 2D-RGO (e^−^) + O_2_ → (˙O_2_^−^)(˙O_2_^−^) + MO → CO_2_ + H_2_Og-C_3_N_4_ (h^+^) + MO → CO_2_ + H_2_O

During this process, N=N bond of MO was broken under the attack of generated radicals, and aromatic hydrocarbons such as 4-amino-*N*,*N*-dimethylaniline and 4-diazenyl-*N*,*N*-dimethyl benzenamine were produced. Then the aromatic rings were continuously opened under the attack of reactive oxygen species and converted into carbon dioxide and water eventually.^28–30^

## 4. Conclusion

In summary, ternary RGO/g-C_3_N_4_/LaCO_3_OH composite was constructed by coupling RGO with heterostructured g-C_3_N_4_/LaCO_3_OH through a hydrothermal reaction, and serve as an effective catalyst for the photodegradation of MO under visible light. Compared with pure g-C_3_N_4_, the MO photodegradation rate can be increased from 0.0083 min^−1^ to 0.0150 min^−1^ by constructing g-C_3_N_4_/LaCO_3_OH heterojunction, and further boosted to 0.0326 min^−1^ by introducing RGO as the cocatalyst, which is 3.93 times higher than pure g-C_3_N_4_. Such significant improvement is owing to the improved photocatalytic activities stemmed from the combination of g-C_3_N_4_/LaCO_3_OH heterostructure with RGO, which can accelerate the transfer and restrict the recombination of the photogenerated carriers, and prolong their lifetime. This study realized the utilization of LaCO_3_OH with a broad bandgap in the visible light region as a promising photocatalyst for the MO degradation, and also proposed a novel strategy for the further development of photocatalyst based on semiconductors with wide bandgaps.

## Conflicts of interest

There are no conflicts to declare.

## Supplementary Material

RA-013-D3RA02415F-s001
