# Supplementary material for: The construction of a heterostructured RGO/g-C3N4/LaCO3OH composite with enhanced visible light photocatalytic activity for MO degradation
Source: RSC Adv. 2023 May 19;13(22):15302–10. doi: 10.1039/d3ra02415f (PMC10196886; doi:10.1039/d3ra02415f)
Supplement: RA-013-D3RA02415F-s001 [file RA-013-D3RA02415F-s001.pdf]

Electronic Supplementary Material (ESI) for RSC Advances.
This journal is © The Royal Society of Chemistry 2023

The construction of heterostructured RGO/g-C_3N_4/$LaCO_3OH$ composite with enhanced visible light photocatalytic activity for MO degradation

Deng Gu,[a,b] Yuanjin Wang,[a] Zhiman Liang,[a] Yanting Dou,[a] Zhenhe Xu,[*a] Jiqi Zheng,[*a] Yaguang Sun,[b] Fu Ding[*b] and Yu Gao[*a]

[a]College of Environmental and Chemical Engineering, Dalian University, Dalian 116622, China.

[b]Key Laboratory of Inorganic Molecule-Based Chemistry of Liaoning Province, Shenyang University of Chemical Technology, Shenyang, 110142, China.

*Correspondence: xuzh@syuct.edu.cn (Z. Xu); jiqizheng@yeah.net (J. Zheng); dingfu@syuct.edu.cn (F. Ding); gaoy777@126.com (Y. Gao)

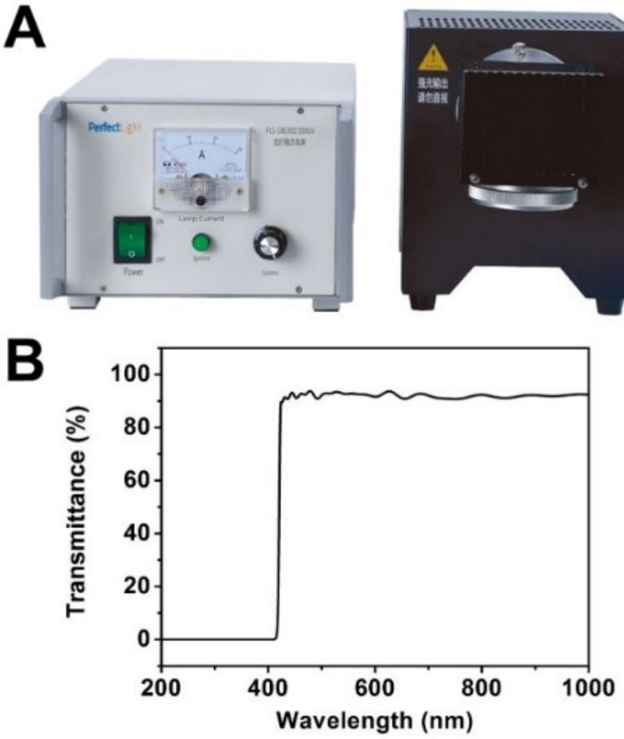

Figure S1. (A) The photograph of the 300 W xenon lamp (PLS-SXE 300, Beijing Perfect Light Co., Ltd.) and (B) the transmission spectrum of the filtered light.

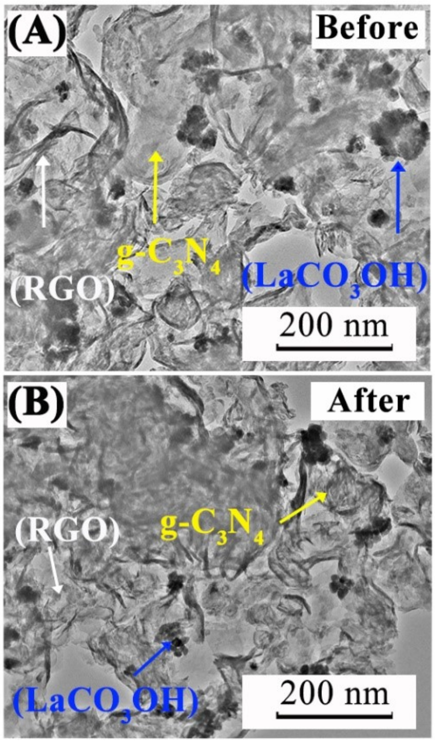

Figure S2. TEM images of RGO/g-C_3N_4/$LaCO_3OH$ before (A) and after (B) the cycling test.
